# Supplementary material for: A Prospective Birth Cohort Study on Maternal Cholesterol Levels and Offspring Attention Deficit Hyperactivity Disorder: New Insight on Sex Differences
Source: Brain Sci. 2017 Dec 23;8(1):3. doi: 10.3390/brainsci8010003 (PMC5789334; doi:10.3390/brainsci8010003)
Supplement: Supplementary file 1 [file brainsci-08-00003-s001.pdf]

A Prospective Birth Cohort Study on Maternal Cholesterol Levels and Offspring Attention Deficit Hyperactivity Disorder: New Insight on Sex Differences: Supplementary Materials

Table S1. ICD-9 and ICD-10 codes for the diagnosis of each neurodevelopmental disorder.

Neurodevelopmental disorder	ICD-9 codes	ICD-10 codes
ASD	299.0, 299.00, 299.01, 299.8, 299.80, 299.81, 299.9, 299.90, 299.91	F84.0, F84.8, F84.9
ADHD	314.0, 314.00, 314.01, 314.1, 314.2, 314.8, 314.9	F90, F90.0, F90.1, F90.2, F90.8, F90.9
Disturbance of conduct	312.0–312.9	F91, F91.0, F91.2, F91.3, F91.8, F91.9
Delays in development	315.0–315.9	F81.0, R48.0, F81.81, F81.2, F81.89, F80.1, F80.2, H93.25, F80.4, F80.81, F80.0, F80.82, F80.89, F82, F88, F81.9, F89
Intellectual disabilities	317-317	F70, F71, F72, F73, F78, F79
Failure to thrive	783.4, 783.40, 783.41, 783.42, 783.43	R62.50, R62.51, R62.0, R62.52
Congenital anomalies	740–759.9	Q00–Q99

Table S2. Maternal and child characteristics for participants excluded and included in the analysis.

**Variable**	**Total, No. (%)**	**Excluded, No. (%)**	**Included, No. (%)**	***p*-value**[ǂ]
Total	3098 (100)	1619 (52.26)	1479 (47.74)	
Maternal Age				0.209
<20	288 (9.30)	140 (8.65)	148 (10.01)	
20–34	2246 (72.50)	1166 (72.02)	1080 (73.02)	
≥35	556 (17.95)	305 (18.84)	251 (16.97)	
Education level				0.844
Below college degree	2642 (85.28)	1364 (84.25)	1278 (86.41)	
College degree or above	420 (13.56)	219 (13.53)	201 (13.59)	
Race ethnicity				<0.001
Black	1965 (63.43)	997 (61.58)	968 (65.45)	
White	227 (7.33)	153 (9.45)	74 (5.00)	
Hispanic	682 (22.01)	325 (20.07)	357 (24.14)	
Others	209 (6.75)	129 (7.97)	80 (5.41)	
Smoking during pregnancy				0.045
Never	2496 (80.57)	1267 (78.26)	1229 (83.10)	
Quitter	238 (7.68)	127 (7.84)	111 (7.51)	
Continuous	330 (10.65)	191 (11.80)	139 (9.40)	
Child's sex				0.181
Female	1529 (49.35)	780 (48.18)	749 (50.64)	
Male	1567 (50.58)	837 (51.70)	730 (49.36)	
Delivery type				0.008
C-section	1116 (36.02)	616 (38.05)	500 (33.81)	
Vaginal	1967 (63.49)	988 (61.03)	979 (66.19)	
Season of child's birth				0.697
Jan to March	721 (23.27)	388 (23.97)	333 (22.52)	
April to June	725 (23.40)	375 (23.16)	350 (23.66)	
July to September	848 (27.37)	446 (27.55)	402 (27.18)	
October to December	802 (25.89)	408 (25.20)	394 (26.64)	
Gestational age, week				<0.001
Mean (SD)	37.6(3.5)	37.2(3.8)	38.1(3.1)	
Birthweight, g				<0.001
Mean (SD)	2898.3(819.7)	2808.3(865.9)	2996.7(754.0)	

[ǂ] The p-values were obtained from chi-square test or t-test between children with and without any ADHD diagnosis.

Table S3. The stratified analysis results on the association between maternal HDL levels (every 20 mg/dL increase) and the risk of ADHD in offspring.

Variable		Beta	SE	P-value
Child's sex	Female	-0.08	0.13	0.563
	Male	-0.28	0.1	0.004
Maternal Age	<20	-0.3	0.26	0.244
	20-34	-0.12	0.09	0.194
	>=35	-0.52	0.2	0.010
Smoking during pregnancy	Never	-0.28	0.09	0.002
	Quitter	-0.04	0.23	0.864
	Continuous	0.018	0.22	0.934
Education level	Below college degree	-0.19	0.08	0.022
	College degree or above	-0.42	0.26	0.111
Race ethnicity	Black	-0.17	0.09	0.064
	White	-0.23	0.39	0.561
	Hispanic	-0.32	0.17	0.065
	Others	0.103	0.48	0.831
Parity	Nulliparous	-0.04	0.12	0.764
	Multiparous	-0.32	0.1	0.002
Intrauterine infection	No	-0.24	0.08	0.005
	Yes	-0.02	0.23	0.920
Delivery type	C-section	0.04	0.13	0.758
	Vaginal	-0.34	0.1	0.001
Gestational age and birthweight	Term birth & normal birthweight	-0.26	0.1	0.009
	Term birth & low birthweight	0.239	0.29	0.413
	Preterm birth & normal birthweight	-0.46	0.27	0.092
	Preterm birth & low birthweight	-0.05	0.18	0.783

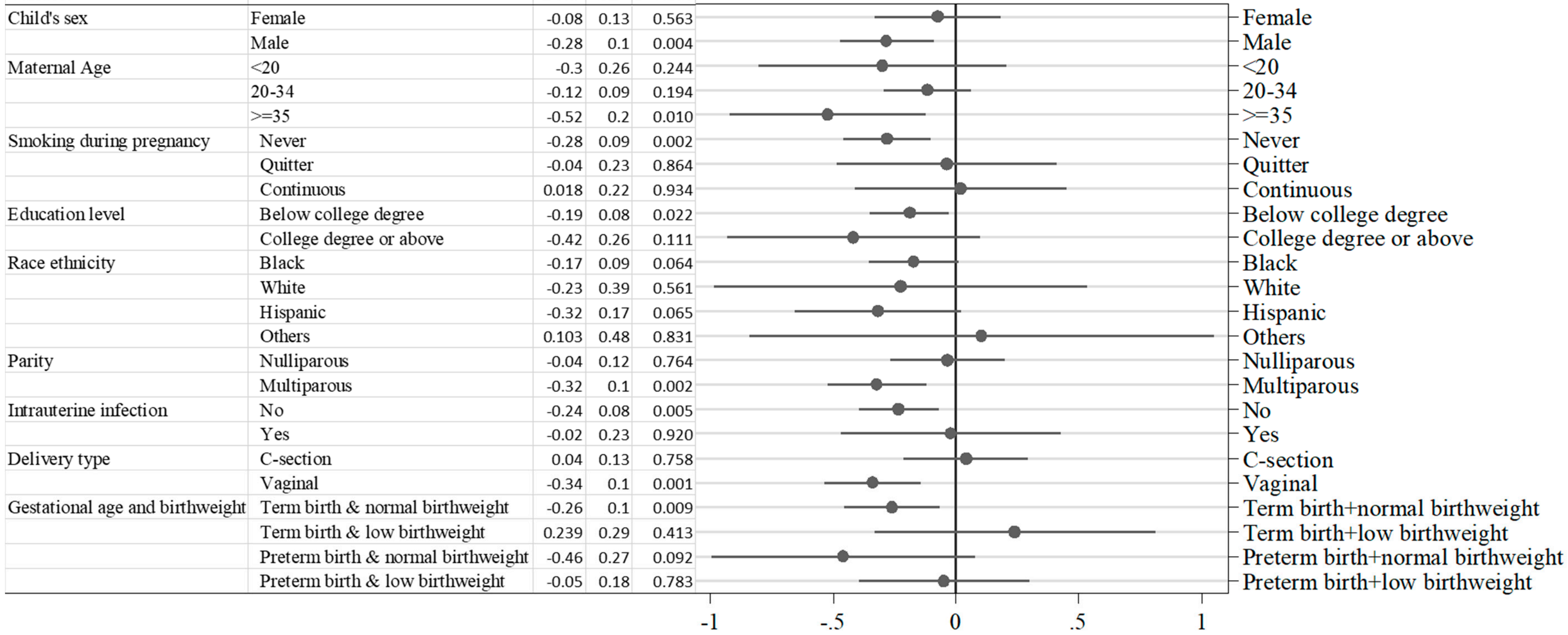

Covariates included maternal age at delivery, maternal race/ethnicity, maternal education, smoking during pregnancy, intrauterine infection, parity, baby's gender, mode of delivery, preterm birth, birthweight.

Table S4. The joint association of child's gender and maternal HDL levels with the risk of any specialist ADHD diagnosis.

Gender	Maternal HDL Level	ADHD, No. (%)	NT, No. (%)	OR	95% CI		*p*-value
Female		59 (8.16)	664 (91.84)	1.00			
Male		155 (23.24)	512 (76.76)	3.26	2.35	4.53	<0.001
	Maternal HDL effects within gender						
Female	Q4	9 (4.59)	187 (95.41)	1.00			
	Q3	19 (9.95)	172 (90.05)	2.24	0.98	5.16	0.057
	Q2	17 (10.83)	140 (89.17)	2.59	1.10	6.09	0.029
	Q1	14 (7.82)	165 (92.18)	1.65	0.68	3.99	0.266
Male	Q4	29 (19.86)	117 (80.14)	1.00			
	Q3	28 (17.50)	132 (82.50)	0.80	0.44	1.46	0.474
	Q2	48 (24.24)	150 (75.76)	1.21	0.70	2.07	0.494
	Q1	50 (30.67)	113 (69.33)	1.65	0.95	2.86	0.073
	Joint effects of maternal HDL and gender						
Female	> median	28 (7.24)	359 (92.76)	1.00			
	≤ median	31 (9.23)	305 (90.77)	1.24	0.72	2.12	0.440
Male	> median	57 (18.63)	249 (81.37)	2.87	1.77	4.67	<0.001
	≤ median	98 (27.15)	263 (72.85)	4.44	2.81	7.02	<0.001

NT was defined as free of any neurodevelopmental disorder diagnosis; ADHD was defined as any specialist ADHD diagnosis; covariates included maternal age at delivery, maternal race/ethnicity, maternal education, smoking during pregnancy, intrauterine infection, parity, baby's gender, mode of delivery, preterm birth, birthweight.

Table S5. The joint association of child's gender and maternal TG levels with the risk of any specialist ADHD diagnosis.

Gender	Maternal TG Level	ADHD, No.(%)	NT, No.(%)	OR	95% CI		*p*-value
Female		59 (8.16)	664 (91.84)	1.00			
Male		155 (23.24)	512 (76.76)	3.31	2.39	4.59	<0.001
	Maternal TG effects within gender						
Female	Q1	14 (8.05)	160 (91.95)	1.24	0.55	2.77	0.605
	Q2	13 (7.26)	166 (92.74)	1.00			
	Q3	13 (7.30)	165 (92.70)	1.01	0.45	2.27	0.984
	Q4	19 (9.90)	173 (90.10)	1.50	0.68	3.31	0.310
Male	Q1	45 (26.16)	127 (73.84)	1.54	0.90	2.65	0.116
	Q2	30 (18.63)	131 (81.37)	1.00			
	Q3	40 (24.10)	126 (75.90)	1.39	0.80	2.42	0.242
	Q4	40 (23.81)	128 (76.19)	1.40	0.79	2.49	0.245
	Joint effects of maternal TG and gender						
Female	Q2	13 (7.26)	166 (92.74)	1.00			
	Q1, Q3, Q4	46 (8.46)	498 (91.54)	1.17	0.61	2.24	0.631
Male	Q2	30 (18.63)	131 (81.37)	2.87	1.43	5.76	0.003
	Q1, Q3, Q4	125 (24.70)	381 (75.30)	4.04	2.20	7.41	<0.001

NT was defined as free of any neurodevelopmental disorder diagnosis; ADHD was defined as any specialist ADHD diagnosis; covariates included maternal age at delivery, maternal race/ethnicity, maternal education, smoking during pregnancy, intrauterine infection, parity, baby's gender, mode of delivery, preterm birth, birthweight.

Table S6. The joint association of child's gender and maternal HDL levels with the risk of any ADHD diagnosis (last diagnosis older than six years).

Gender	Maternal HDL Level	ADHD, No.(%)	NT, No.(%)	OR	95% CI		*p*-value
Female		51 (7.13)	664 (92.87)	1.00			
Male		132 (20.50)	512 (79.50)	3.22	2.27	4.57	<0.001
	Maternal HDL effects within gender						
Female	Q4	8 (4.10)	187 (95.90)	1.00			
	Q3	17 (8.99)	172 (91.01)	2.19	0.91	5.27	0.081
	Q2	14 (9.09)	140 (90.91)	2.30	0.92	5.76	0.075
	Q1	12 (6.78)	165 (93.22)	1.49	0.58	3.83	0.411
Male	Q4	23 (16.43)	117 (83.57)	1.00			
	Q3	24 (15.38)	132 (84.62)	0.85	0.44	1.61	0.612
	Q2	40 (21.05)	150 (78.95)	1.23	0.68	2.20	0.492
	Q1	45 (28.48)	113 (71.52)	1.83	1.02	3.31	0.043
	Joint effects of maternal HDL and gender						
Female	> median	25 (6.51)	359 (93.49)	1.00			
	≤ median	26 (7.85)	305 (92.15)	1.14	0.64	2.03	0.662
Male	> median	47 (15.88)	249 (84.12)	2.63	1.57	4.41	<0.001
	≤ median	85 (24.43)	263 (75.57)	4.26	2.63	6.90	<0.001

NT was defined as free of any neurodevelopmental disorder diagnosis; ADHD was defined as any ADHD diagnosis; covariates included maternal age at delivery, maternal race/ethnicity, maternal education, smoking during pregnancy, intrauterine infection, parity, baby's gender, mode of delivery, preterm birth, birthweight.

Table S7. The joint association of child's gender and maternal TG levels with the risk of any ADHD diagnosis (last diagnosis older than six years).

Gender	Maternal TG Level	ADHD, No.(%)	NT, No.(%)	OR	95% CI		*p*-value
Female		51 (7.13)	664 (92.87)	1.00			
Male		132 (20.50)	512 (79.50)	3.25	2.30	4.61	<0.001
	Maternal TG effects within gender						
Female	Q1	12 (6.98)	160 (93.02)	1.16	0.49	2.74	0.727
	Q2	12 (6.74)	166 (93.26)	1.00			
	Q3	11 (6.25)	165 (93.75)	0.90	0.38	2.14	0.817
	Q4	16 (8.47)	173 (91.53)	1.29	0.56	2.98	0.546
Male	Q1	40 (23.95)	127 (76.05)	1.71	0.96	3.05	0.070
	Q2	24 (15.48)	131 (84.52)	1.00			
	Q3	31 (19.75)	126 (80.25)	1.32	0.72	2.43	0.371
	Q4	37 (22.42)	128 (77.58)	1.58	0.85	2.91	0.146
	Joint effects of maternal TG and gender						
Female	Q2	12 (6.74)	166 (93.26)	1.00			
	Q1, Q3, Q4	39 (7.26)	498 (92.74)	1.05	0.53	2.07	0.892
Male	Q2	24 (15.48)	131 (84.52)	2.48	1.18	5.20	0.016
	Q1, Q3, Q4	108 (22.09)	381 (77.91)	3.68	1.95	6.93	<0.001

NT was defined as free of any neurodevelopmental disorder diagnosis; ADHD was defined as any ADHD diagnosis; covariates included maternal age at delivery, maternal race/ethnicity, maternal education, smoking during pregnancy, intrauterine infection, parity, baby's gender, mode of delivery, preterm birth, birthweight.

Table S8. The association between maternal cholesterol and the risk of other neurodevelopmental disorders in offspring.

**Maternal cholesterols**		**OtherDD, No. (%)**	**NT, No. (%)**	**Crude OR**	**95% CI**		***p*-value**	**Adjusted OR**	**95% CI**		***p*-value**
HDL clinical cut-off	≥ 50 mg/dL	444 (33.08)	898 (66.92)	1.00				1.00			
	< 50 mg/dL	158 (36.24)	278 (63.76)	1.15	0.92	1.44	0.227	1.13	0.90	1.43	0.300
HDL quartiles	Q4 (>73 mg/dL)	142 (31.84)	304 (68.16)	1.00				1.00			
	Q3 (61–73 mg/dL)	164 (35.04)	304 (64.96)	1.15	0.88	1.52	0.305	1.06	0.80	1.41	0.664
	Q2 (50–60 mg/dL)	138 (32.24)	290 (67.76)	1.02	0.77	1.35	0.898	0.90	0.67	1.20	0.465
	Q1 (< 50 mg/dL)	158 (36.24)	278 (63.76)	1.22	0.92	1.61	0.168	1.12	0.83	1.49	0.461
HDL binary	> median (60 mg/dL)	306 (33.48)	608 (66.52)	1.00				1.00			
	≤ median (60 mg/dL)	296 (34.26)	568 (65.74)	1.04	0.85	1.26	0.728	0.97	0.79	1.19	0.764
HDL linear trend (every 20 mg/dL increase)		602 (33.86)	1176 (66.14)	0.95	0.85	1.06	0.391	0.99	0.89	1.12	0.933
TG clinical cut-off	< 200 mg/dL	388 (34.28)	744 (65.72)	1.00				1.00			
	≥ 200 mg/dL	214 (33.13)	432 (66.87)	0.95	0.77	1.17	0.623	1.08	0.86	1.35	0.500
TG quartiles	Q1 (<135 mg/dL)	154 (34.92)	287 (65.08)	1.00				1.00			
	Q2 (135–176 mg/dL)	159 (34.87)	297 (65.13)	1.00	0.76	1.31	0.987	1.04	0.78	1.37	0.806
	Q3 (177–232 mg/dL)	154 (34.61)	291 (65.39)	0.99	0.75	1.30	0.922	1.04	0.78	1.40	0.766
	Q4 (>232 mg/dL)	135 (30.96)	301 (69.04)	0.84	0.63	1.11	0.213	0.96	0.71	1.31	0.801
TG binary	Q2	159 (34.87)	297 (65.13)	1.00				1.00			
	Q1, Q3, Q4	443 (33.51)	879 (66.49)	0.94	0.75	1.18	0.597	0.97	0.77	1.22	0.788
TG linear trend (every 20 mg/dL increase)		602 (33.86)	1176 (66.14)	1.00	0.97	1.02	0.747	1.01	0.99	1.04	0.303
LDL quartiles	Q1 (<96 mg/dL)	156 (35.62)	282 (64.38)	1.00				1.00			
	Q2 (96–121 mg/dL)	157 (35.36)	287 (64.64)	0.99	0.75	1.30	0.937	1.00	0.75	1.32	0.983
	Q3 (122–150 mg/dL)	155 (33.99)	301 (66.01)	0.93	0.71	1.23	0.610	0.98	0.73	1.30	0.873
	Q4 (>150 mg/dL)	134 (30.45)	306 (69.55)	0.79	0.60	1.05	0.104	0.85	0.63	1.14	0.278
LDL linear trend (every 20 mg/dL increase)		602 (33.86)	1176 (66.14)	0.95	0.90	0.99	0.024	0.96	0.91	1.01	0.102
TC quartiles	Q1 (<176 mg/dL)	150 (34.72)	282 (65.28)	1.00				1.00			
	Q2 (176–214 mg/dL)	164 (36.36)	287 (63.64)	1.07	0.82	1.42	0.611	1.13	0.85	1.51	0.385

Q3 215–254 mg/dL)	160 (35.56)	290 (64.44)	1.04	0.79	1.37	0.796	1.11	0.83	1.48	0.478
Q4 (>254 mg/dL)	128 (28.76)	317 (71.24)	0.76	0.57	1.01	0.058	0.85	0.62	1.15	0.282
TC linear trend (every 20 mg/dL increase)	602 (33.86)	1176 (66.14)	0.97	0.93	1.00	0.035	0.98	0.94	1.01	0.213

NT was defined as without any neurodevelopmental disorder diagnosis; otherDD is defined as any neurodevelopmental disorder diagnosis other than ADHD; the multiple logistic regression model was adjusted for maternal age at delivery, maternal race/ethnicity, maternal education, smoking during pregnancy, intrauterine infection, parity, child's sex, mode of delivery, preterm birth, and birthweight.
